# Stable interindividual differences in modafinil’s effect on vigilance during sleep deprivation

**DOI:** 10.3389/fphar.2025.1607444

**Published:** 2025-09-25

**Authors:** Jeroen Van Cutsem, Martine Van Puyvelde, Nicholas H. Van den Berg, Emilie Dessy, Frederic Detaille, An Van Rompay, Olivier Mairesse, Catherine Drogou, Fabien Sauvet, Xavier Neyt, Nathalie Pattyn, Guido Simonelli

**Affiliations:** ^1^ VIPER Research Unit, Royal Military Academy, Brussels, Belgium; ^2^ Human Physiology and Sports Physiotherapy Research Group, Vrije Universiteit Brussel, Brussels, Belgium; ^3^ Experimental and Applied Psychology, Department of Psychology and Educational Sciences, Vrije Universiteit Brussel, Brussels, Belgium; ^4^ Clinical and Lifespan Psychology, Department of Psychology and Educational Sciences, Vrije Universiteit Brussel, Brussels, Belgium; ^5^ Centre de Recherche Avancée en Médecine du Sommeil, Centre Intégré Universitaire De Santé et De Services Sociaux Du Nord-de-l’ile-de-Montréal, Montréal, QC, Canada; ^6^ Service Public de Wallonie, Namur, Belgium; ^7^ Aviation Safety Directorate, Brussels, Belgium; ^8^ Military Hospital Queen Astrid, Brussels, Belgium; ^9^ Brain, Body and Cognition, Department of Psychology, Faculty of Psychology and Educational Sciences, Vrije Universiteit Brussel, Brussels, Belgium; ^10^ Institut de Recherche Biomédicale des Armées (IRBA), Bretigny sur Orge, France; ^11^ UMR VIFASOM, université Paris-cité, Paris, France; ^12^ Département de Médecine, Université de Montréal, Montreal, QC, Canada; ^13^ Département de Neuroscience, Université de Montréal, Montreal, QC, Canada

**Keywords:** psychostimulant, aviation, overconfidence, sleep deprivation, fatigue

## Abstract

**Rationale:**

In specific operational contexts (i.e., military aviation), the off-label use of modafinil is officially regulated. However, safety concerns are still raised.

**Objectives:**

To study the stability and robustness of interindividual differences in modafinil sensitivity, both in terms of risks and benefits in military student pilots.

**Methods:**

Eleven healthy military student pilots (21 ± 2 yr; 1 woman) were tested in a within-subject randomized counterbalanced crossover design to compare modafinil (2 × 200 mg; EXP) vs. placebo (CON) effects during extended wakefulness (24 h). Throughout both trials, participant’s vital signs, mood, vigilance [i.e., Psychomotor Vigilance Task (PVT)] and self-monitoring ability were measured. Additionally, four participants were genotyped [i.e., COMT (rs4680) and PER3 (rs228697)]. We used Pearson correlation coefficients to evaluate the relationship between PVT performance and the performance self-monitoring scores. To evaluate the stability of interindividual differences in the effectiveness of modafinil to improve PVT performance and sleepiness, an intraclass correlation coefficient (ICC) was calculated for the delta score (CON-EXP) of both outcome measures.

**Results:**

Modafinil significantly improved PVT performance (p ≤ 0.034) and sleepiness (p ≤ 0.029) at 2a.m. and 4a.m. during the sleep deprivation night. The stability of the non-adjusted reaction time-delta score was very high (ICC = 0.90). Non-adjusted reaction time only correlated with the performance self-monitoring scores in CON (r ≥ −0.35; p < 0.001).

**Conclusion:**

Stable interindividual differences in the effectiveness of modafinil to counteract the sleep deprivation-associated decrease in vigilance exist. Further research should focus on quantifying the extent to which modafinil-induced overconfidence and subjective rebound sleepiness actually constitute potential problems in operational environments (e.g., perhaps using war game simulations).

## 1 Introduction

Modafinil (2-[(diphenyl-methyl)-sulfinyl]acetamide) is a centrally active alpha 1 adrenergic agonist that, from its discovery onwards (i.e., in 1987), has demonstrated promising applications both in healthy (e.g., military context) and pathological (e.g., narcolepsy and hypersomnia) populations (Billiard and Broughton, 2018; Lyons and French, 1991; Ooi et al., 2019), specifically in a sleep deprived context. On the extent and nature of the cognitive effects of modafinil in healthy, non-sleep-deprived humans there has been little consensus (Battleday and Brem, 2015). Nonetheless, in a sleep deprived situation, it triggered interest in a military context when its vigilance-increasing effects were found to parallel d-amphetamine, while preserving a user-safe profile (Billiard and Broughton, 2018; Lyons and French, 1991). Subsequently, following preliminary testing (Lagarde et al., 1995), the French army allowed military physicians accompanying the 1991-Daguet operation and the 1991-Operation Desert Storm to prescribe modafinil for sleep-restricted military pilots and mechanics (Billiard and Broughton, 2018). Meanwhile, the applications of modafinil were under assessment in pathological populations, and by 1992, modafinil was officially registered for the treatment of narcolepsy in France, becoming commercially available in 1994 (Billiard and Broughton, 2018).

To date, the use of modafinil is still only approved for the treatment of excessive sleepiness associated with narcolepsy and the mechanisms underlying its wakefulness-promoting and cognitive effects are still partially unknown (Delli Pizzi et al., 2025). However, modafinil is nevertheless widely used in the absence of a disease (off-label use) to counteract the negative effects of lack of sleep, and to improve attentional effort, alertness, focus and/or mental stamina (Cross et al., 2020; Ooi et al., 2019; Teodorini et al., 2020; Van Puyvelde et al., 2022). Off-label use of modafinil seems to induce minimal and minor side effects, for example, headache and diarrhea (Cross et al., 2020; Ooi et al., 2019; Teodorini et al., 2020). Moreover, in specific situations, off-label use of modafinil can be officially regulated. Among healthy American (Caldwell et al., 2009), Indian (Pandit, 2016), Singaporean (Ooi et al., 2019) and Dutch (Wingelaar-Jagt et al., 2022a) military pilots, as well as within the Canadian Space Agency (Thirsk et al., 2009), modafinil is legally distributed in situations where pilots are already sleep-deprived but operational needs requires them to continue their mission.

Despite the widespread off-label use of modafinil and the minimal occurrence of side effects, safety concerns are still raised (Cesta et al., 2020; Cid-Jofré et al., 2020; Van Puyvelde et al., 2022) which challenge the current notion that the benefits of modafinil use outweigh the disadvantages (Teodorini et al., 2020). For example, Cid-Jofré et al. (2020) recently demonstrated that chronic (i.e., 14 days of 75 mg/kg/day) modafinil administration during preadolescence in rats impairs dopaminergic neurotransmission in the nucleus accumbens, and decreases the capacity of rats to perceive rewarding effects of social play. Despite the difference in treatment-dose (i.e., in humans, modafinil-dosing usually ranges between 200 and 400 mg/day; in an individual of 70 kg this equals a maximum dose of 5.7 mg/kg/day), this finding should raise questions on potential adverse effects or long-term impacts of modafinil use. Furthermore, in pregnant women, Damkier and Broe (2020) reported that first-trimester *in utero* exposure to modafinil was associated with an increased risk of congenital malformations compared to control. However, this finding was later challenged by the results of Cesta et al. (2020). In addition, regulatory agencies have also raised concerns about hypersensitivity reactions and neuropsychiatric adverse effects (Peñaloza et al., 2013).

The risk-benefit assessment of modafinil use has also been reconsidered in the military context. In their recent review, Van Puyvelde et al. (2022) concluded that, despite the usefulness of modafinil in operational sleep-deprived contexts, its use as a performance-enhancing stimulant and smart drug (i.e., outside of an occasional sleep deprivation constraint) should be banned in military contexts given the current gaps in knowledge and the uncertainty surrounding its risk–benefit profile. To substantiate this statement, the authors highlighted that the potential risks for abuse, and overconfidence in its capabilities, should be thoroughly investigated in both regular and occasional use. Indeed, Baranski & Pigeau (1997) were the first to investigate the effect of modafinil on self-monitoring ability, demonstrating that the use of modafinil results in overconfidence particularly during the first few hours following administration. Moreover, their follow-up research showed that even in non-sleep-deprived individuals, a trend towards mild overconfidence following modafinil use is present (Baranski et al., 2004). Accordingly the warning that modafinil use comes with the cost of overconfidence still stands (Colzato et al., 2021).

Taken together, the results above highlight the need for additional research on the risk-benefit profile for regulated off-label use of modafinil. Similar to other stimulants [e.g., caffeine (Carswell et al., 2020)], sensitivity to modafinil seems to vary between individuals (Bodenmann and Landolt, 2010; Bodenmann et al., 2009a; Bodenmann et al., 2009b). Moreover, these interindividual differences in response to modafinil use are replicable and robust (Bodenmann et al., 2009a; Caldwell et al., 2020), and thus appear to be trait-like and dependent on an individual’s genotype (Van Dongen et al., 2005). For example, a specific genotype of the gene that encodes the catechol-O-methyltransferase (COMT) protein has already been linked to the effect of modafinil on sleep-wake regulation (Bodenmann and Landolt, 2010; Bodenmann et al., 2009b; Bodenmann et al., 2009a). The COMT protein is an important breakdown enzyme of cortical catecholamines (e.g., dopamine), and a functional Valine158Methionine single-nucleotide polymorphism (SNP) in the COMT gene (NCBI SNP-ID: rs4680) is known to impact both the enzymatic activity and the amount of the COMT protein (Chen et al., 2004). In addition to the COMT gene genotype, a Proline864Alanine SNP in the Period Circadian Regulator 3 (PER3) gene (NCBI SNP-ID: rs228697) has been linked to diurnal preference (e.g., being a morning lark or an evening owl), sleep disorders (Hida et al., 2014), and to intra-individual variation in athletic performance and effort with time of day (Anderson et al., 2018). The PER3 protein plays a role in the loop that keeps the suprachiasmatic nucleus synchronized with a 24-h rhythm, and a functional Proline864Alanine polymorphism in the PER3 gene could alter the secondary structure and/or the phosphorylation of the PER3 protein (Hida et al., 2014). The PER3 SNP has been found to influence PVT lapses performance and sleepiness during an extended wakefulness paradigm (Erblang et al., 2021) and, as such, could set up specific individuals to benefit more from modafinil use compared to others.

Genetically-mediated risk-benefit profiling is one way to individualize modafinil use and optimize the off-label use of modafinil in regulated situations. As demonstrated above, off-label modafinil use is most regulated in the military context. The strategic use of stimulants during military operations can be valuable in specific situations, such as when pilots have had a night of disturbed sleep prior to a planned work shift, when changing from a night flight schedule to a day flight schedule, or when performing flights which cross multiple time zones.

Overall, the main aim of the present study was to confirm, in military student pilots, the existence of stable and robust interindividual differences in modafinil sensitivity, both in terms of risks and benefits. To do so, physiological, psychological, and behavioral measures that are associated with sleep-wake regulation, cognitive performance, self-monitoring, and the occurrence of known side effects were followed up in an extended wakefulness paradigm of 24-h. Military student pilots were followed up throughout the extended wakefulness period, and during the 2 days and nights following the extended wakefulness period. Based on the results of Bodenmann and Landolt (2010) and Bodenmann et al. (2009) we hypothesized that the twice-daily consumption of 200 mg of modafinil (i.e., 200 mg at 8p.m. of day 1 and 200 mg at 4p.m. of day 2 of the extended wakefulness paradigm) would, in modafinil sensitive individuals, counteract the known vigilance decrement and subjective sleepiness associated with extended wakefulness. Similarly, in terms of modafinil-use associated risks, we hypothesized that eventual side effects and sleep recovery impairments would occur in an individualized way.

## 2 Methods

### 2.1 Participants and ethical approval

An *a priori* sample size calculation based on the results reported in the study of Caldwell et al. (2020) (reported effect size n_p_^2^ of condition × time interaction in terms of Psychomotor Vigilance Task (PVT) performance = 0.378) was used to determine that 10 participants were needed. To ensure sufficient data in case of dropout, eleven military student pilots—who have been carefully screened and selected based on rigorous medical (EASA-compliant Class 1 exam) and cognitive (a bachelor’s degree, preferably in science, technology, engineering or mathematics and a dedicated psychological evaluation) criteria to start their military pilot training—were included in the present study (1 woman, 10 men; age = 21 ± 2 yr). Each participant gave written informed consent before the study and had no history of modafinil use. This study was conducted in direct response to operational needs from the Belgian Air Force, and the experimental protocol and procedures were approved by the Institutional Review Board of CHU/UVC Brugmann (REF: CE 2021/191), Belgium. All participants were given written instructions describing all procedures related to the study but were naïve regarding its aims and hypotheses. To avoid a large confounding placebo effect based on expectations, participants were informed that the purpose of the study was to investigate the effect of two potentially performance-enhancing substances: modafinil and slow-release caffeine (as per the approved protocol). There was no slow-release caffeine administration but a placebo condition (calcium lactate administration). Participants were individually debriefed after completing all trials, to explain the reason for this deception and to inform them of their personal results.

### 2.2 Experimental design

This study was a randomized single-blinded, placebo-controlled, counterbalanced cross-over design and took place while certain COVID-19 restrictions were still in place. During the period of the study implementation, Belgium was 3 weeks into phase 3 of its deconfinement plan. Phase 3 aimed to reintroduce personal freedoms—such as limited social interactions and mobility—while keeping serious safety constraints in place to prevent a resurgence of the virus (e.g., group gatherings were capped at 10 people, safety distance rules (1.5 m) applied except within households, restaurants, cafés, hotels, and night shops reopened under clear protocols and internal travel within Belgium was permitted). Due to the remaining safety constraints, ethical approval was obtained for all trials to take place in the home environment of each participant and data was gathered by making use of the online experiment builder “Gorilla,” as well as virtual consultations with a flight surgeon to measure vitals. This data collection was part of the military testing protocol to officially regulate modafinil use in Belgian operational pilots on deployment.

Participants were asked to complete two interventional trials (i.e., modafinil-trial and calcium lactate-trial) that were separated by a 10-day washout period and performed in a randomized counterbalanced order. Modafinil exhibits a terminal half-life of 9–14 h with peak blood concentrations 2–4 h after absorption, and should therefore be eliminated from the human body within four and a half days (i.e., 99% eliminated) (Wong et al., 1998). We instructed the participants to refrain from alcohol, caffeine and any other psychostimulant use while participating in the study. The day before each experimental condition, the participants were expected to sleep according to their normal sleep pattern and avoid the practice of vigorous physical activity. In addition, participants were asked to have a similar meal and cognitive load (e.g., perform the same kind of activities) on day 1 of both the modafinil-trial, from now on referred to as EXP, and the calcium lactate-trial, from now on referred to as CON (e.g., gaming on both day 1 of EXP and CON, or making a puzzle on both day 1 of EXP and CON). The use of any kind of medicinal products during and between the trials was prohibited. If participants could not meet these standards, they were excluded from the study.

**FIGURE 1:**
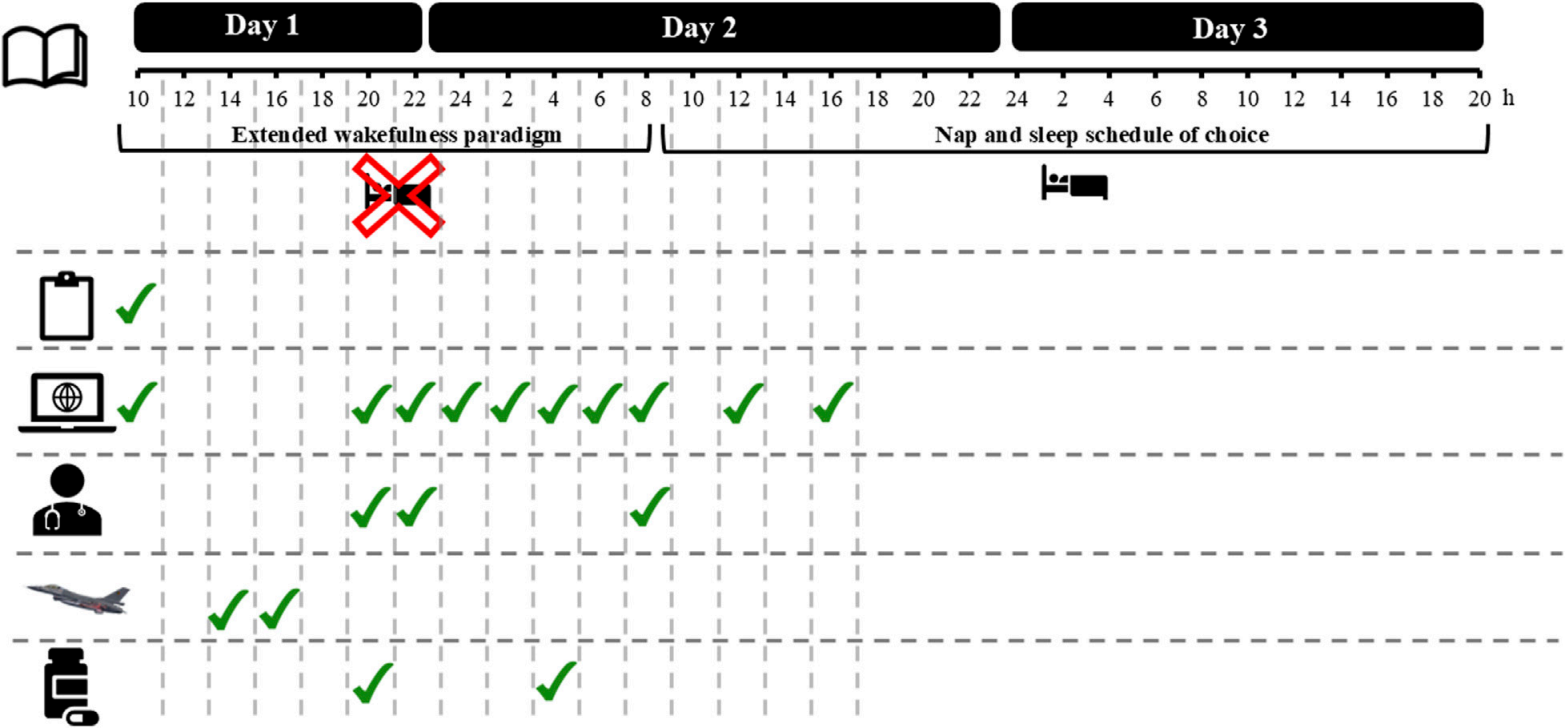
Overview of a 3-day trial. 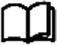 = sleep diary; 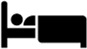 = sleep deprivation protocol; 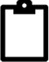 = baseline descriptives; 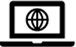 = online test protocol; 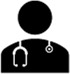 = virtual meeting with the medical doctor; 
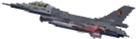
 = virtual gaming session; 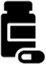 = administration protocol.

Both in EXP and CON, the protocol took 3 days each to complete (see Figure 1 and Materials for more information), and participants were blinded to the intervention. Before starting a trial, compliance with instructions was assessed with a checklist. Around 10a.m. on day 1 of both EXP and CON, participants first performed a 60-min familiarization online test protocol to get acquainted with the online testing environment, the questionnaires, and to mitigate learning effects in the cognitive task. To perform an online test protocol, participants were asked to isolate themselves from their housemates in a sound-deprived room in their home environment with as little distractors present as possible (i.e., no time indicators, no smartphone) and to be seated in a comfortable chair. Following on the familiarization online test protocol, at 2p.m., participants started a 4-h flight simulation session (www.flightgear.org). After completing the flight simulation session, participants reported themselves online in a virtual meeting at 8p.m. with the responsible flight surgeon and the principal investigator (JVC), to 1) consume their first two 100-mg tablets (the second batch of tablets was consumed at 4p.m. on day 2); 2) check their vital signs; 3) begin their extended wakefulness paradigm until 8a.m. on day 2; and 4) start-up their second (the first is the familiarization online test protocol) of 10 online test protocols to evaluate the impact of modafinil on physiological, psychological and behavioral measures that are associated with sleep-wake regulation, cognitive performance, self-monitoring and the occurrence of known side effects. Once the second online test protocol was started, participants logged off from the virtual meeting with the flight surgeon and the principal investigator. Following on the second online test protocol, the remaining 8 online test protocols had to be performed at fixed time intervals on day one and two (see Figure 1). As previously mentioned, for each online test protocol participants had to isolate themselves from their housemates in a sound-deprived room in their home environment (i.e., take place in their own room and close the door). In between online test protocols, participants were free to do what they wanted, but were instructed to not take in any alcohol-containing or caffeine-containing drinks and to not perform any training at a high intensity (i.e., going for a jog was not a problem, but no fatiguing exercise such as a 10-km run at maximal speed). Besides the online test protocols, the participants were also requested to fill out a wake and sleep diary on the day when the extended wakefulness paradigm started, and on the day after their first night of normal sleep, to evaluate a possible impact of modafinil on recovery sleep. Given that this study was performed with COVID-related restrictions still in place, this study was designed as a full online study with medical supervision provided online as well. Throughout both EXP and CON, the flight surgeon held, besides the first virtual meeting, two more virtual meetings to follow-up any side effects and provide medical supervision—one at 10p.m. of day 1 and one at 8:30a.m. of day 2. Both the flight surgeon as well as the principal investigator were also constantly available to answer any questions from study participants.

### 2.3 Protocol-specifics

#### 2.3.1 Extended wakefulness paradigm

Participants had to stay awake during the first night of the 3-day trial in both EXP and CON. Specifically, participants were required to avoid sleeping until after the last virtual meeting (i.e., 8:30a.m. day 2). Once the last virtual meeting was completed, participants were made aware again that, aside from the timepoints that an online test protocol had to be performed, they could take naps whenever they wanted and could adopt a sleep schedule of their choice. Before starting the extended wakefulness paradigm, participants were instructed that they should stay mentally and/or physically active during the night of day 1, as the goal was not to engage in only online activity (e.g., binge-watching tv shows) throughout the night. To monitor how participants followed these instructions, they were encouraged to share photos of their activities in a dedicated group chat.

#### 2.3.2 Online test protocols

In terms of the online test protocol, participants had to perform this protocol multiple times (i.e., 10 times; Day 1: 10a.m., 8p.m., 10p.m. and 12p.m.; Day 2: 2a.m., 4a.m., 6a.m., 8a.m., 12a.m. and 4p.m.; See Figure 1). All online test protocols were identical to one another and encompassed a medical questionnaire to evaluate the participants’ vital signs, followed by multiple visual analog scales (VAS), a flight safety question, the Profile of Mood States (POMS), a prospective subjective prediction of PVT performance, a Psychomotor Vigilance Task (PVT), a retrospective subjective evaluation of PVT performance, and the National Aeronautics and Space Administration Task Load Index (NASA-TLX). Online test protocols were designed in the online experiment builder ‘Gorilla’ (https://gorilla.sc/).

#### 2.3.3 Administration protocol

A modafinil (Provigil) and placebo (calcium lactate; Sterop) administration protocol was performed during one night of extended wakefulness, separated by a washout period of 10 days. Like the study of Baranski et al. (1998) a preventive administration protocol was used that includes two 200-mg doses separated by an 8-h interval. A 200-mg dose has been shown to maximize the benefits of modafinil use while minimizing the risk of unwanted side effects (Van Puyvelde et al., 2022). Both in EXP (modafinil) and CON (placebo), participants were instructed to take in four tablets throughout the night of sleep deprivation. The first two tablets had to be taken at 8p.m. of day 1, while the last two tablets were taken at 4a.m. of day 2. Each Provigil tablet weighed 100 mg and contained 100 mg modafinil, equating to a total dose of 400 mg of modafinil. Each placebo tablet weighed 300 mg and contained 39 mg of elemental calcium per tablet, equating to a total dose of 156 mg. To assess the successfulness of the participant-blinding method, participants were asked, after completion of all trials and before the debriefing (i.e., participants did not know yet that they had taken a placebo instead of a slow-release caffeine tablet), to guess when they took modafinil and when slow-release caffeine (i.e., placebo).

### 2.4 Dependent variables

#### 2.4.1 Vital signs

Participants were asked to self-assess their vital signs guided by a medical questionnaire, and to add this information on their online medical questionnaire at each test timepoint of the protocol. Basic physiological parameters included heart rate (Rossmax AC1000F), blood pressure (Rossmax AC1000F), body temperature (home-available thermometer) and respiration rate (self-count in a 30-s time interval). In addition, they were asked to report whether they had perceived any side effects, and if so, what side effects they had perceived (i.e., headache, palpitations, hyperventilation, diarrhea, anxiety, nervousness, allergic reaction, insomnia and/or other).

#### 2.4.2 Visual analog scales (VAS)

Participants were asked to complete six VAS: a mental fatigue-VAS, a physical fatigue-VAS, a sleepiness-VAS, an anxiety-VAS, a stress-VAS and a motivation-VAS. To provide clarity about this outcome measures, a definition of fatigue, sleepiness and stress was given as follows. Fatigue was defined as “*A feeling of exhaustion (being mentally or physically empty). Fatigue is essentially countered by rest*”, sleepiness was defined as “*Characterized by difficulty staying awake or alert. Sleepiness is primarily countered by sleep*” and stress was defined as “*A feeling of physical or mental tension caused by physical, cognitive, environmental or emotional factors*”. Minimum and maximum descriptors were; “*no mental fatigue*”, ‘*intense mental fatigue*’; “*no physical fatigue*”, “*intense physical fatigue*”; “*really alert*”, “*extremely sleepy*”; “*no anxiety*”, “*extreme anxiety*”; “*no stress*”, “*extreme stress*”; “*completely not*”, “*extremely*”.

#### 2.4.3 Flight safety

Following the VAS, participants were also asked whether they perceived themselves to be safe to fly an aircraft (“Yes” or “No”).

#### 2.4.4 Profile of mood states

The 37-item POMS - SF (Curran et al., 1995) contains six scales which assess different states of mood: anxiety, depression, anger, vigor, fatigue, and confusion (disappointment). Each of the scales is measured using 5 to 8 items on a Likert scale from 0 to 4. Each state is measured on a scale of 0–4, which is calculated by summing the scores of the items in each state and dividing that score by the number of items.

#### 2.4.5 Prospective subjective prediction of PVT performance

Right before (i.e., prospectively) performing the PVT, participants were asked to predict their own performance. They were asked to answer, on a 100-mm VAS, the following question “*Estimate how well you think you will perform in the PVT (taking into account both your reaction time as well as your accuracy)*”, from “*poorly*” to “*extremely well*”.

#### 2.4.6 Psychomotor Vigilance Task

The PVT (Dinges and Powell, 1985) measures the reaction time to a given stimulus by pressing the space bar when the stimulus appears (i.e., a measure of vigilance), and the lapses when the stimulus is missed, or when reaction time exceeds 500 ms. Participants were instructed to place the index of their dominant hand on the space bar and to keep it there throughout the PVT. The visual stimulus appeared at random intervals (2, 4, 6, 8 or 10 s) and the task lasted for 10 min (i.e., 20 blocks of ∼30 s, in each block of 30 s all inter-stimulus intervals were used in a random order). Optimal reaction times were between 200 and 400 ms (i.e., optimal response domain). To assess performance on the PVT, both adjusted (i.e., only taking into account the responses within the optimal response domain), and non-adjusted reaction time (RT) were taken into account, as well as accuracy (ACC; i.e., percentage of responses within optimal response domain), and lapses (i.e., responses 500 ms after stimulus presentation). Before the start of each PVT, participants were given five practice stimuli to warm up.

#### 2.4.7 Retrospective subjective evaluation of PVT performance

Immediately after (i.e., retrospectively) performing the PVT, participants were asked to rate their own performance. They were asked to answer, on a 100-mm VAS, the following question: “*E. how well you think you performed in the PVT (taking into account both your reaction time as well as your accuracy)*”, from “*poorly*” to “*extremely well*”.

#### 2.4.8 National Aeronautics and Space Administration Task Load Index

The NASA-TLX (Hart and Staveland, 1988) is composed of six subscales (i.e., mental demand, physical demand, temporal demand, performance, effort and frustration) that are rated on a 100-points range with 5-point steps. As such, all NASA-TLX subscales were scored on a scale from 0–21. It was applied to evaluate the subjective workload of the PVT.

#### 2.4.9 Sleep diary

A wake and sleep diary (Monk et al., 1994) was used regarding sleep (completed by participants the morning upon waking) and daytime activity (completed prior to their bedtime). The participants had to indicate, on a 24-h scale with a time resolution of 15 min, 1) when they started trying to fall asleep, 2) when they actually fell asleep, 3) whether they woke up after falling asleep and, if yes, for how long, 4) when they woke up, and 5) whether they took a nap out of bed and, if yes, for how long. Subsequently, the following outcome measures were calculated: total time spent in bed, sleep onset latency, wake after sleep onset, total sleep time, total nap time, bedtime and risetime. In addition, participants were also asked to rate their sleep quality, quality of the wake-up and shape of the day (score on a 10-point scale).

### 2.5 Independent variables

#### 2.5.1 Subjective sleep quality

The Pittsburgh Sleep Quality Index (PSQI) (Buysse et al., 1989) includes 19 self-assessment items that combine to give 7 components of the overall score, with each component receiving a score of 0–3. A score of 0 indicates no difficulty, while a score of 3 indicates severe difficulty. The 7 components of the score add up to give an overall score ranging from 0 to 21 points, with 0 indicating no difficulty and 21 indicating major difficulties. An overall sum of 5 or more indicates a ‘bad’ sleeper.

#### 2.5.2 Chronotype

The participant’s chronotype was assessed via a 16-item questionnaire developed by Oginska et al. (2017) and via the Single-Item Chronotyping (SIC) scale, developed by Putilov et al. (2021). In the chronotype questionnaire developed by Oginska et al. (2017), participants must indicate whether they agree with statements; to do so, they have four-answer options: “yes”, “rather yes”, “rather no” and “no”. Two dimensions of the chronotype are assessed using this questionnaire: the morningness-eveningness scale (i.e., ME; 8 items) and the subjective amplitude scale (i.e., AM; 8 items). The first scale is used to evaluate the preferred time of day for different activities (example item: I find I think better in the morning). The second scale is the distinction scale and investigates the subjective sense of distinction of daily changes (i.e., the amplitude or range of diurnal fluctuations). Regarding the SIC-scale, participants are prompted to choose their chronotype from six simple charts depicting different levels of their activity (high or moderate or low) at three different intervals of the day (morning, daytime, and evening). The six charts that are included in the SIC represent: 1 = morning type; 2 = evening type; 3 = daytime sleepy type; 4 = daytime type; 5 = highly active type and 6 = moderately active type.

#### 2.5.3 COMT and PER3 genotyping

Four participants agreed to be genotyped for the Valine 158 Methionine SNP of COMT (NCBI SNP-ID: rs4680) and for the Proline864Alanine SNP of PER3 (NCBI SNP-ID: rs228697). To genotype, the recently developed loop-mediated isothermal amplification and melting curve analysis (LAMP-MC) technique was used (Drogou et al., 2020). Venous blood samples were collected in EDTA tubes and subsequently aliquoted in sterile microtubes and stored at −20 °C until LAMP-MC analysis. LAMP-MC assays were realized with the customized Human Sleep Deprivation Combo kit (Cat#LC-SDC-LP-24, LaCAR MDX, Liège, Belgium). See Drogou et al. (2020) for further detailed information.

For the COMT gene genotype, if at codon 158 in the COMT gene the base letter G is substituted by an A, this variant is called ‘A allele’. If this is not the case, this variant is called ‘G allele’. Everyone has two copies of the COMT gene (i.e., two alleles), and as such, the possible codon 158-COMT gene genotypes are: G/G, A/A and G/A. For the PER3 gene genotype, if at codon 864 in the PER3 gene, the base letter C is substituted by a G, this variant is called ‘G allele’. If this is not the case, this variant is called ‘C allele’. The possible codon 864-PER3 gene genotypes are: C/C, G/G and C/G.

### 2.6 Statistical analysis

All data are presented as mean ± SD unless stated otherwise. A small amount of data (i.e., 1.8%) was randomly missing (e.g., due to connectivity problems). Subsequently, to avoid the listwise exclusion of a participant due to a missing value, missing values were replaced by the mean of that variable at that specific time interval. The method of replacing missing values by the mean was chosen due to the percentage of missing values being very low. Therefore, the probability that this method altered the statistical outcome is marginally low (Field, 2009).

The Shapiro–Wilk test was used to test the normality of the data. If data were not normally distributed (i.e., POMS depression, confusion, tension, anger and fatigue subscale, anxiety-VAS, stress-VAS, NASA-TLX physical load, temporal load and frustration subscale, heart rate, baseline sleep onset latency, wake after sleep onset, total sleep time, quality of the wake-up and napping time, first night sleep onset latency, total sleep time, sleep efficiency, subjective sleep quality, quality of the wake-up, shape of the day, napping time, occurrence rate of side effects and safe to fly), non-parametric Wilcoxon tests were used to observe the effect of condition (EXP vs. CON). All other parameters were normally distributed or normally distributed after a square root transformation (i.e., ACC was subtracted from a constant factor [100.1] and subsequently square root transformed; number of lapses was square root transformed). The effect of condition and time on all normally distributed parameters was assessed by a two-way (2 × 10) repeated-measures ANOVA. Sphericity was verified by the Mauchly’s test. When the assumption of sphericity was not met, the Greenhouse-Geisser correction was applied to correctly interpret main and interaction effects. If significant interaction effects were observed, subsequent paired-samples t-tests and one-way repeated measures ANOVAs were performed to, respectively, elucidate the main effect of condition in each time interval (two-sided p-values are included in the results section) and the main effect of time in each condition. To interpret the results of follow-up testing, the Bonferroni correction was applied.

To assess the accuracy of both the prospective and retrospective performance prediction and evaluation, a Pearson correlation coefficient was calculated with the overall PVT performance measure (i.e., non-adjusted RT). The benchmarks suggested by Akoglu (2018) were used to interpret the strength of the resulting correlation coefficients.

The stability of interindividual differences for the delta score (CON-EXP) of non-adjusted RT and sleepiness was quantified with the intraclass correlation coefficient (ICC), which was calculated as the between-subjects variance divided by the sum of the between- and within-subject variances. A delta score was calculated to numerically express the condition effect. For further information on this statistical analysis see Tucker et al. (2007). In short, by experimental design, in the current protocol we observed significant variations in both mood (e.g., perceived sleepiness) and presence of daylight. Consequently, ICC values can be interpreted as evidence of both stability (across day and night) and robustness (across a varying psychological state), and provides a direct assessment of the proportion of variance in the data explained by inter-individual variability (Van Dongen et al., 2004).

A role for the included independent variables (i.e., subjective sleep quality and chronotype) in modafinil effectiveness was evaluated by dividing participants in two groups. Participants were stratified once based on PSQI score (group A = PSQI score <5; group B = PSQI score ≥5; 5 is the threshold that is used in clinical practice to define a ‘bad’ sleeper), and once based on chronotype (group A = SIC chronotype profile 5; group B = all other SIC chronotype profiles; i.e., the highly active type vs. all other types). Subsequently, a possible difference between groups was statistically evaluated with an independent samples t-test.

Significance was set at <0.05 for all analyses, which were conducted using the Statistical Package for the Social Sciences, version 28 (SPSS Inc., Chicago, IL).

## 3 Results

### 3.1 PVT performance

**FIGURE 2:**
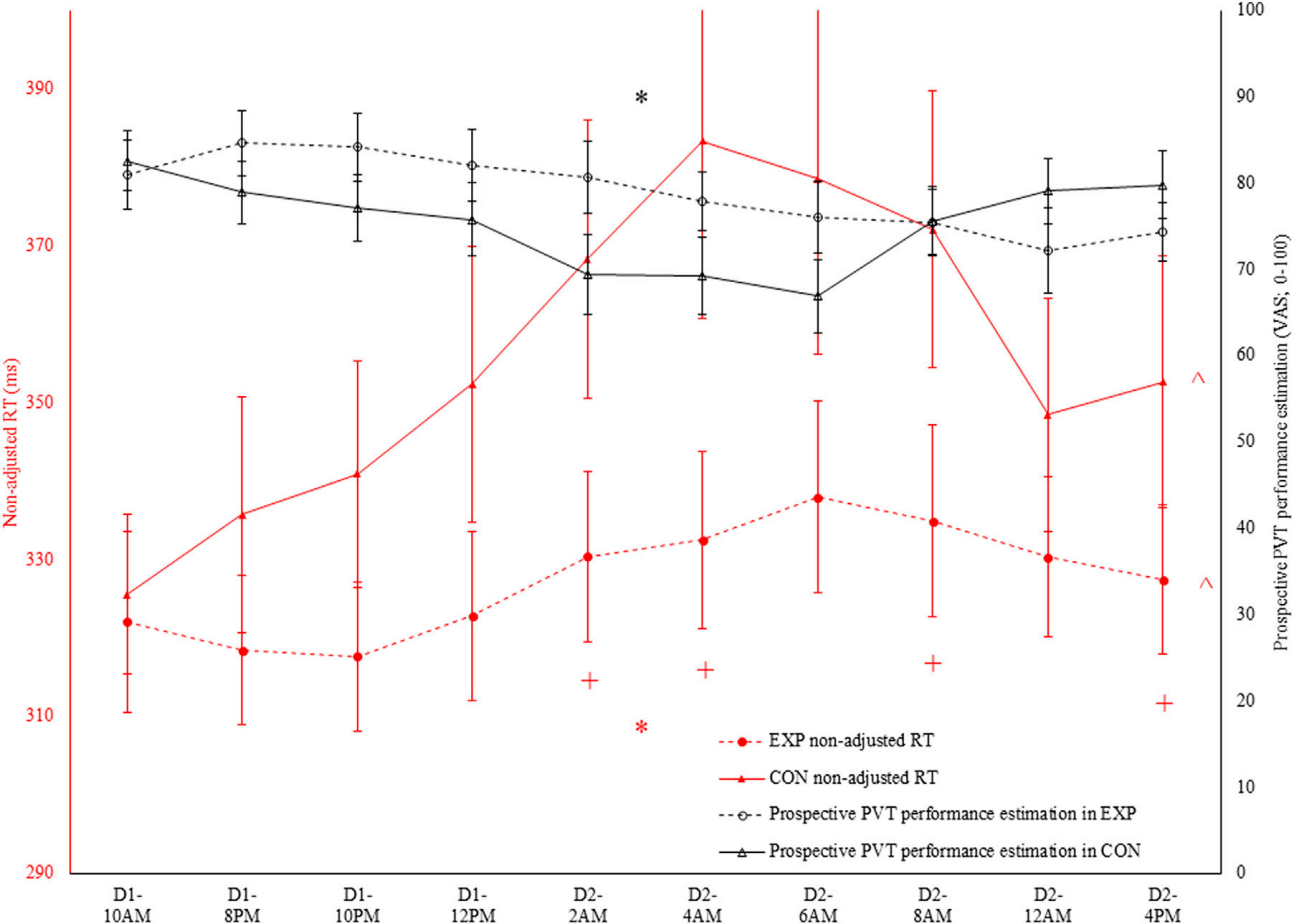
In black: prospective Psychomotor Vigilance Task (PVT) performance prediction (VAS; 0–100) throughout both the experimental (EXP) and control condition (CON). * denotes a significant interaction effect between condition and time (p < 0.05). In red: non-adjusted reaction time (RT) in the PVT task throughout both EXP and CON. * denotes a significant interaction effect between condition and time (p < 0.05). ^ denotes a significant main effect of time (p < 0.05). + denotes a significant difference between EXP and CON at that specific time (p < 0.05).

**FIGURE 3:**
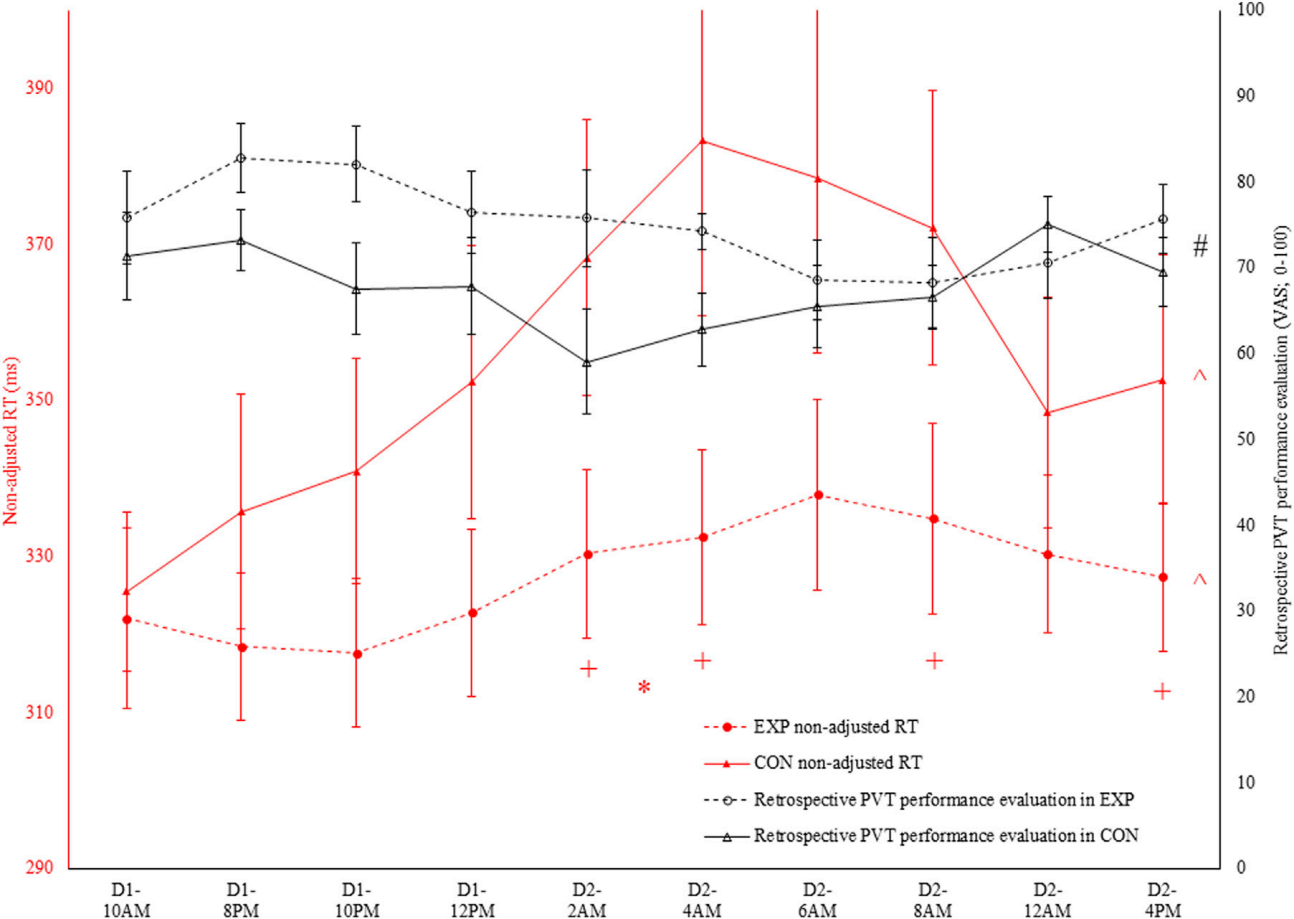
In black: retrospective Psychomotor Vigilance Task (PVT) performance evaluation [visual analog scale (VAS); 0–100] throughout both the experimental (EXP) and control condition (CON). ^#^ denotes a significant main effect of condition (p < 0.05). In red: non-adjusted reaction time (RT) in the PVT task throughout both EXP and CON.

There was an interaction effect between condition and time for non-adjusted RT (see Figures 2, 3) and adjusted RT, ACC and lapses (p ≤ 0.032; η_p_^2^ ≥ 0.20; See Table 1). For all four PVT outcomes, performance was significantly better in EXP compared to CON. This was the case at 2a.m. (p ≤ 0.034), 4a.m. (p ≤ 0.022) and 4p.m. (p ≤ 0.050). Specifically for non-adjusted RT, ACC and lapses a significant difference was also observed at 8a.m. (p ≤ 0.044). In terms of the effect of time, all four PVT outcomes were significantly impaired in time in CON (p ≤ 0.006; higher RT coupled to lower ACC; see Figures 2, 3). In EXP, only non-adjusted RT (see Figures 2, 3) and adjusted RT were significantly impaired in time (p ≤ 0.028).

**TABLE 1:** Psychomotor Vigilance Task performance in both the experimental (EXP) and control condition (CON; mean ± SD).

	Day 1				Day 2					
	10AM	8PM	10PM	12PM	2AM	4AM	6AM	8AM	12AM	4PM
*EXP*										
Non-adj RT (ms)*	322 ± 31	318 ± 32	318 ± 36	323 ± 36	330 ± 37^+^	333 ± 41^+^	338 ± 41	335 ± 34^+^	330 ± 32	327 ± 36^+^
Adj RT (ms)*	308 ± 22	308 ± 22	306 ± 25	310 ± 27	312 ± 25^+^	315 ± 26^+^	317 ± 24	316 ± 23	314 ± 22	310 ± 22^+^
ACC (%)*	91 ± 8	91 ± 11	91 ± 10	89 ± 11	87 ± 11^+^	87 ± 14^+^	84 ± 18	87 ± 11^+^	88 ± 10	88 ± 12^+^
Lapses*	2 ± 2	1 ± 2	1 ± 2	2 ± 2	2 ± 3^+^	3 ± 3^+^	3 ± 3	3 ± 3^+^	2 ± 2	2 ± 3^+^
CON										
Non-adj RT (ms)*	326 ± 34	336 ± 50	341 ± 48	352 ± 58	368 ± 59^+^	383 ± 75^+^	379 ± 74	372 ± 58^+^	348 ± 49	353 ± 53^+^
Adj RT (ms)*	307 ± 17	315 ± 28	319 ± 25	324 ± 28	330 ± 28^+^	331 ± 32^+^	330 ± 27	328 ± 23	321 ± 26	322 ± 27^+^
ACC (%)*	88 ± 12	86 ± 19	86 ± 14	80 ± 22	65 ± 31^+^	67 ± 29^+^	69 ± 27	72 ± 22^+^	79 ± 20	79 ± 20^+^
Lapses*	3 ± 5	4 ± 7	3 ± 6	5 ± 8	9 ± 11^+^	12 ± 13^+^	13 ± 18	9 ± 9^+^	4 ± 6	7 ± 9^+^

* denotes a significant interaction effect between condition and time (p < 0.05). + denotes a significant difference between EXP and CON at that specific time (p < 0.05). Non-adj RT = non-adjusted reaction time; Adj RT = adjusted reaction time; ACC = accuracy.

### 3.2 Mood and workload

The POMS-questionnaire depression-subscale was higher (p = 0.042) at baseline in EXP (0.18 ± 0.23) compared to CON (0.05 ± 0.1), while fatigue-subscale scores were significantly different at 2a.m. during the extended wakefulness paradigm (p = 0.021) and at 12a.m. after the extended wakefulness paradigm (p = 0.045). At 2a.m., the fatigue score was higher in CON (0.65 ± 0.51) compared to in EXP (0.22 ± 0.28), while at 12a.m. this was higher in EXP (0.73 ± 0.52) compared to CON (0.40 ± 0.36). In addition, the vigor-subscale score was higher in EXP compared to CON at both 4a.m. (EXP = 1.42 ± 0.87; CON = 0.91 ± 0.95) and 6a.m. (EXP = 1.61 ± 1.12; CON = 1.05 ± 0.84) during the sleep deprivation night (p ≤ 0.040). Regarding the anger, confusion and tension subscales, no differences between EXP and CON were observed.

Table 2 summarizes all results from the Visual Analog Scales. A significant interaction between condition and time was present for sleepiness (F (3.6, 36.1) = 5.4; p = 0.002; η_p_^2^ = 0.35). Participants perceived a higher degree of sleepiness in the 2a.m. (p = 0.002) and 4a.m. (p = 0.029) time interval in CON compared to EXP. In contrast, at 12a.m. (p = 0.018) and 4p.m. (p = 0.019), a higher amount of sleepiness was perceived in EXP compared to CON. Motivation did not differ between EXP and CON, but it did drop in time (main effect of time; F (9, 90) = 2.1; p = 0.039; η_p_^2^ = 0.17). Participants perceived more stress (p = 0.028) at baseline in EXP compared to CON. Regarding mental fatigue, physical fatigue and anxiety no differences were observed between EXP and CON (see Table 2).

**TABLE 2:** Visual analog scales evaluating mood-related variables in both the experimental (EXP) and control condition (CON; mean ± SD).

	Day 1				Day 2					
	10AM	8PM	10PM	12PM	2AM	4AM	6AM	8AM	12AM	4PM
*EXP*										
Sleepiness*	14 ± 11	21 ± 18	17 ± 19	18 ± 19	21 ± 18^+^	20 ± 10^+^	23 ± 21	26 ± 19	32 ± 24^+^	27 ± 21^+^
Motivation^	78 ± 27	69 ± 28	78 ± 17	72 ± 28	76 ± 21	75 ± 19	72 ± 20	72 ± 19	74 ± 24	70 ± 19
Stress	10 ± 16^+^	6 ± 11	5 ± 11	6 ± 12	4 ± 8	4 ± 8	5 ± 9	7 ± 12	7 ± 11	7 ± 12
Mental fatigue	22 ± 28	22 ± 22	20 ± 22	19 ± 22	20 ± 17	22 ± 10	26 ± 17	26 ± 16	29 ± 20	25 ± 21
Physical fatigue	11 ± 17	15 ± 18	10 ± 15	11 ± 22	12 ± 17	13 ± 12	15 ± 15	18 ± 19	20 ± 17	17 ± 16
Anxiety	5 ± 12	7 ± 16	6 ± 18	5 ± 16	5 ± 13	4 ± 9	4 ± 9	5 ± 12	5 ± 12	3 ± 6
CON										
Sleepiness*	16 ± 17	16 ± 16	21 ± 24	26 ± 21	37 ± 24^+^	36 ± 24^+^	34 ± 19	34 ± 24	21 ± 14^+^	17 ± 15^+^
Motivation^	82 ± 13	83 ± 10	83 ± 11	73 ± 18	68 ± 15	68 ± 17	68 ± 15	69 ± 21	67 ± 20	71 ± 17
Stress	3 ± 7^+^	5 ± 9	6 ± 11	3 ± 9	4 ± 7	3 ± 8	2 ± 6	3 ± 5	3 ± 4	3 ± 6
Mental fatigue	12 ± 10	17 ± 13	19 ± 18	21 ± 18	29 ± 19	30 ± 19	29 ± 16	28 ± 13	20 ± 15	17 ± 15
Physical fatigue	13 ± 15	13 ± 12	14 ± 13	16 ± 15	21 ± 17	16 ± 13	20 ± 15	23 ± 17	13 ± 10	11 ± 11
Anxiety	2 ± 5	5 ± 11	4 ± 11	3 ± 11	3 ± 6	4 ± 10	0 ± 0	2 ± 4	2 ± 4	2 ± 4

* denotes a significant interaction effect between condition and time (p < 0.05). ^ denotes a significant main effect of time (p < 0.05). ^+^ denotes a significant difference between EXP and CON at that specific time (p < 0.05).

In terms of mental, physical, temporal demand, and effort associated with the PVT, no differences were observed between conditions on the NASA TLX outcomes. In contrast, performance-wise, participants perceived that their performance in EXP (15.5 ± 3.7) was superior to in CON (13.7 ± 4.1; main effect of condition; F (1, 10) = 5.6; p = 0.040; η_p_^2^ = 0.36) and that it decreased in time (Baseline = 15.9 ± 4.8; 4p.m. = 14 ± 3.5; main effect of time; F (9, 90) = 2.4; p = 0.019; η_p_^2^ = 0.19). In addition, the frustration-subscale indicated that the participants experienced more frustration in EXP (3.6 ± 2.6) compared to in CON (1.5 ± 1.6; p = 0.011) at 12a.m.

### 3.3 PVT performance subjective prediction and evaluation

Regarding the PVT performance prediction, as depicted in Figure 2, condition and time interacted with one another (F (9, 90) = 4.0; p < 0.001; η_p_^2^ = 0.29). However, participants indicated both in EXP (F (9, 90) = 3.2; p = 0.002; η_p_^2^ = 0.24) and CON (F (3.6, 35.6) = 3.5; p = 0.019; η_p_^2^ = 0.26) that their performance was dropping throughout the sleep deprivation protocol. Follow-up pairwise comparisons did not reveal any significant differences at any time in both EXP and CON. Moreover, paired-samples t-tests did not reveal a significant condition effect at any time. Retrospectively, as depicted in Figure 3, only a main effect of condition was observed (F (1, 10) = 7.9; p = 0.018; η_p_^2^ = 0.44).

**FIGURE 4:**
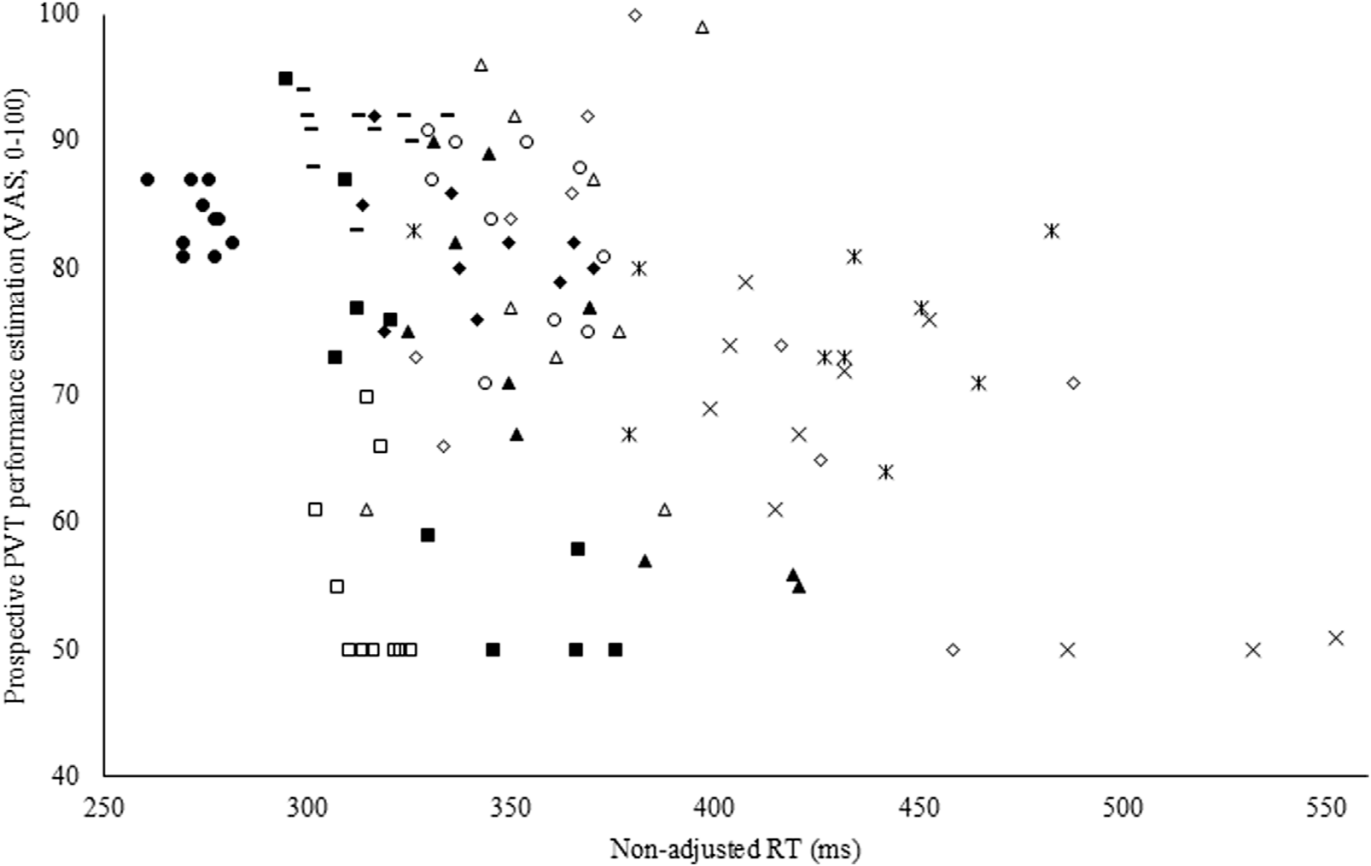
Pearson correlation coefficient between the prospective Psychomotor Vigilance Task (PVT) performance prediction visual analog scale (VAS) and non-adjusted reaction time (RT; r = −0.35; p < 0.001) in the control condition (CON).

**FIGURE 5:**
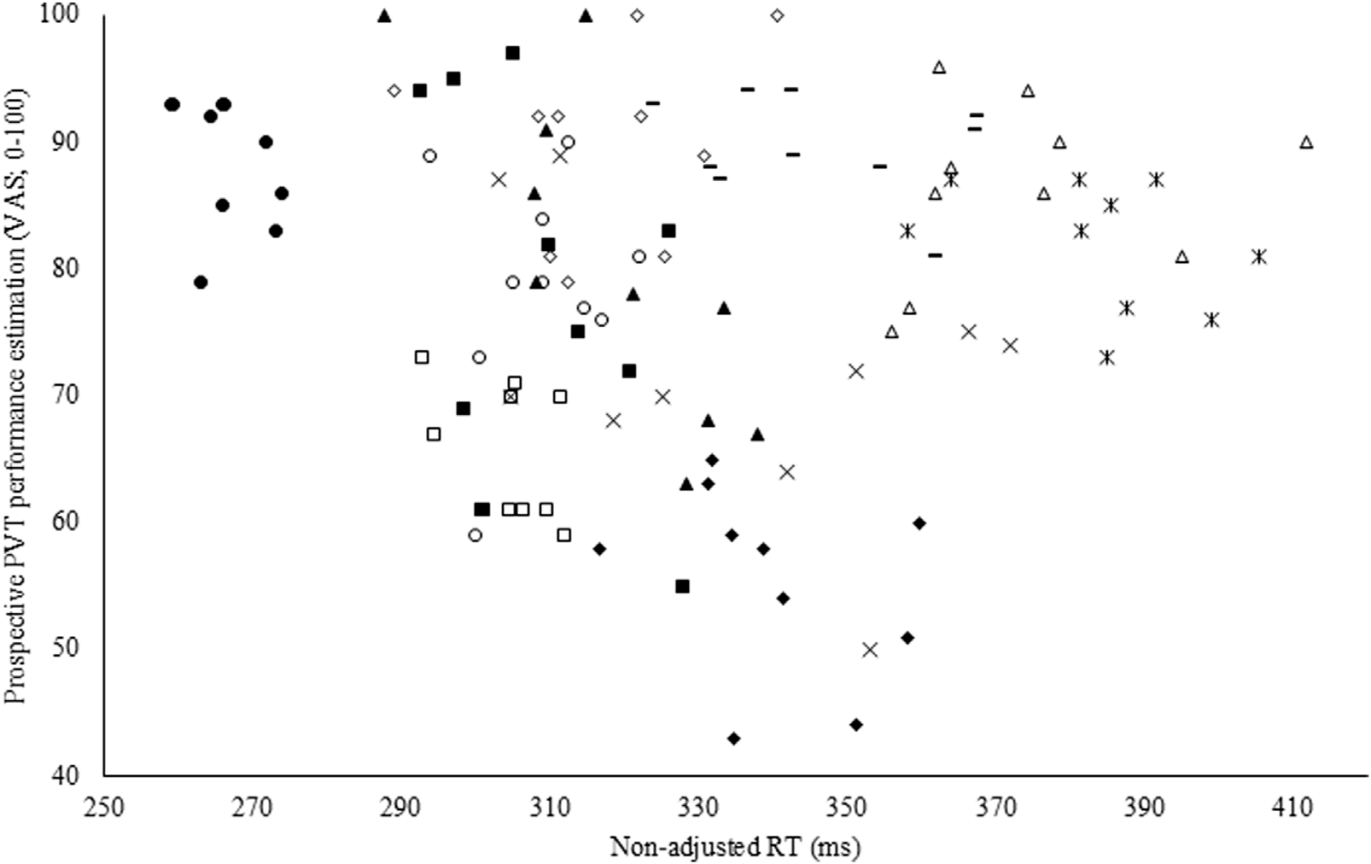
Pearson correlation coefficient between the prospective Psychomotor Vigilance Task (PVT) performance prediction visual analog scale (VAS) and non-adjusted reaction time (RT) in the experimental condition (EXP).

To assess the accuracy of both the subjective performance prediction and evaluation, a Pearson correlation coefficient was calculated with the overall PVT performance measure (i.e., non-adjusted RT). Both prospectively (r = −0.35; p < 0.001; weak; see Figure 4) and retrospectively (r = −0.47; p < 0.001; moderate), non-adjusted RT correlated with the subjective performance prediction and evaluation in CON. However, in EXP, these poor to moderate correlations were absent and thus not statistically significant (see Figure 5).

### 3.4 Vital signs and perceived side effects

Heart rate, blood pressure, body temperature and respiratory rate were self-reported at each online test timepoint. No interaction or main effects of time and condition were observed (see Table 3).

**TABLE 3:** Vital signs in both the experimental (EXP) and control condition (CON; mean ± SD).

	Day 1				Day 2					
	10AM	8PM	10PM	12PM	2AM	4AM	6AM	8AM	12AM	4PM
*EXP*										
HR (bpm)	69 ± 13	66 ± 11	70 ± 10	75 ± 20	73 ± 27	64 ± 9	66 ± 11	75 ± 20	74 ± 19	75 ± 25
Systolic BP (mmHg)	121 ± 10	129 ± 10	131 ± 16	123 ± 17	125 ± 15	127 ± 12	126 ± 18	121 ± 22	122 ± 21	115 ± 15
Diastolic BP (mmHg)	73 ± 10	76 ± 9	79 ± 8	74 ± 7	72 ± 7	72 ± 8	75 ± 11	71 ± 11	74 ± 10	74 ± 8
Tbody (°C)	36.1 ± 0.6	36.2 ± 0.5	36.5 ± 0.6	36.2 ± 0.5	36.2 ± 0.4	36.0 ± 0.5	36.1 ± 0.6	36.2 ± 0.5	36.2 ± 0.5	36.1 ± 0.5
RR (#/min)	12 ± 4	11 ± 2	11 ± 2	11 ± 2	11 ± 3	11 ± 3	11 ± 2	11 ± 3	10 ± 2	11 ± 2
CON										
HR (bpm)	66 ± 9	74 ± 25	79 ± 29	69 ± 19	71 ± 32	62 ± 8	70 ± 21	69 ± 23	68 ± 9	64 ± 6
Systolic BP (mmHg)	121 ± 14	126 ± 24	116 ± 28	122 ± 20	118 ± 16	124 ± 14	118 ± 19	122 ± 26	116 ± 13	123 ± 13
Diastolic BP (mmHg)	70 ± 8	77 ± 12	70 ± 12	70 ± 11	71 ± 8	73 ± 11	70 ± 8	71 ± 12	69 ± 7	69 ± 6
Tbody (°C)	36.3 ± 0.6	36.1 ± 0.9	36.2 ± 0.6	36.2 ± 0.6	35.9 ± 0.5	36.0 ± 0.6	36.0 ± 0.5	36.0 ± 0.7	36.1 ± 0.6	36.3 ± 0.3
RR (#/min)	11 ± 3	11 ± 2	11 ± 2	10 ± 2	10 ± 3	10 ± 2	10 ± 2	10 ± 2	10 ± 2	10 ± 2

HR = heart rate; BP = blood pressure; Tbody = body temperature; RR = respiration rate

In the modafinil condition, five participants experienced side effects (including headache, diarrhea, light nausea, palpitations and nervousness), on average, at 28% of the measurements. In the placebo condition, two participants experienced a headache at 15% of the measurements. Overall, the occurrence rate of these well-known minor side effects was not significantly different between conditions (EXP = 13 ± 18%; CON = 3 ± 6%).

In the modafinil condition, four participants indicated that it was unsafe for them to fly an airplane on one or more occasions (on average, at 20% of the measurements). In the placebo condition, seven participants indicated that it was unsafe for them to fly an airplane on one or more occasions (on average, at 23% of the measurements). Overall, the occurrence rate of being unsafe to fly was not significantly different between conditions (EXP = 7 ± 13%; CON = 15 ± 16%). However, there was a trend towards being more unsafe to fly in CON than in EXP (p = 0.054).

### 3.5 Sleep diary

In terms of self-reported sleep, no baseline differences were found between EXP and CON regarding time spent in bed, sleep onset latency, wake after sleep onset, total sleep time, sleep efficiency, subjective sleep quality, quality of the wake-up, shape of the day and napping time (see Table 4). In addition, no differences between EXP and CON were observed between these specific measures during the first night after having performed the sleep deprivation protocol (see Table 4).

**TABLE 4:** Sleep-related outcomes in both the experimental (EXP) and control condition (CON; mean ± SD).

	Baseline		Night of day 2	
	EXP	CON	EXP	CON
SOL (hr)	0.4 ± 0.2	0.5 ± 0.4	0.5 ± 0.5	0.4 ± 0.3
TIB (hr)	7.1 ± 2.8	7.4 ± 1.9	9.2 ± 1.2	8.1 ± 1.4
TST (hr)	6.4 ± 2.5	6.6 ± 1.7	8.3 ± 1.2	7.5 ± 1.4
WASO (hr)	0.4 ± 0.4	0.3 ± 0.4	0.4 ± 0.4	0.3 ± 0.3
SE (%)	91 ± 5	89 ± 9	90 ± 8	92 ± 6
Subjective sleep quality	7.9 ± 1.5	7.1 ± 1.5	7.9 ± 1.5	8.0 ± 1.1
Quality of the wake-up	6.5 ± 2.0	6.8 ± 1.0	6.4 ± 1.6	7.1 ± 1.1
Shape of the day	7.6 ± 1.0	7.4 ± 0.9	7.3 ± 1.2	7.4 ± 1.4
Napping time (hr)	0.0 ± 0.0	0.1 ± 0.2	0.1 ± 0.3	0.1 ± 0.2

SOL = sleep onset latency; TIB = time in bed; TST = total sleep time; WASO = wake after sleep onset; SE = sleep efficiency

### 3.6 Interindividual differences in modafinil-effectiveness

**FIGURE 6:**
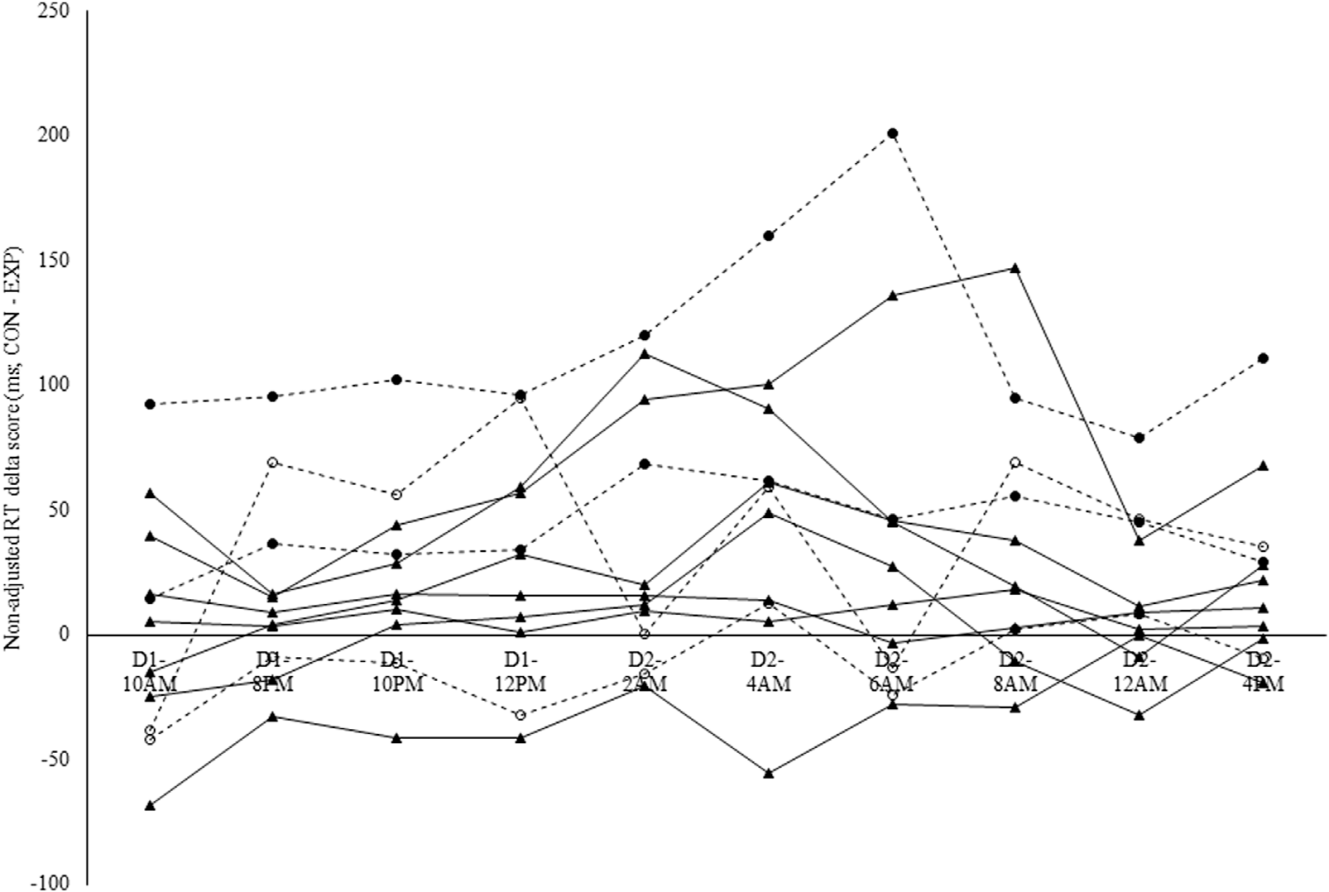
Individual modafinil effectiveness profiles in terms of non-adjusted reaction time (RT) on the Psychomotor Vigilance Task (PVT). Non-adjusted RT delta scores were calculated in ms by subtracting the RT in the experimental condition (EXP-RT) from the RT in the control condition (CON-RT). Each line represents one participant. Dashed lines indicate the participants that were genotyped for the catechol-O-methyltransferase (COMT) and Period Circadian Regulator 3 (PER3) single-nucleotide polymorphism (SNP). Full circles = A/A; Hollow circles = G/A. The intraclass correlation coefficient (ICC) for non-adjusted RT was 0.90.

**FIGURE 7:**
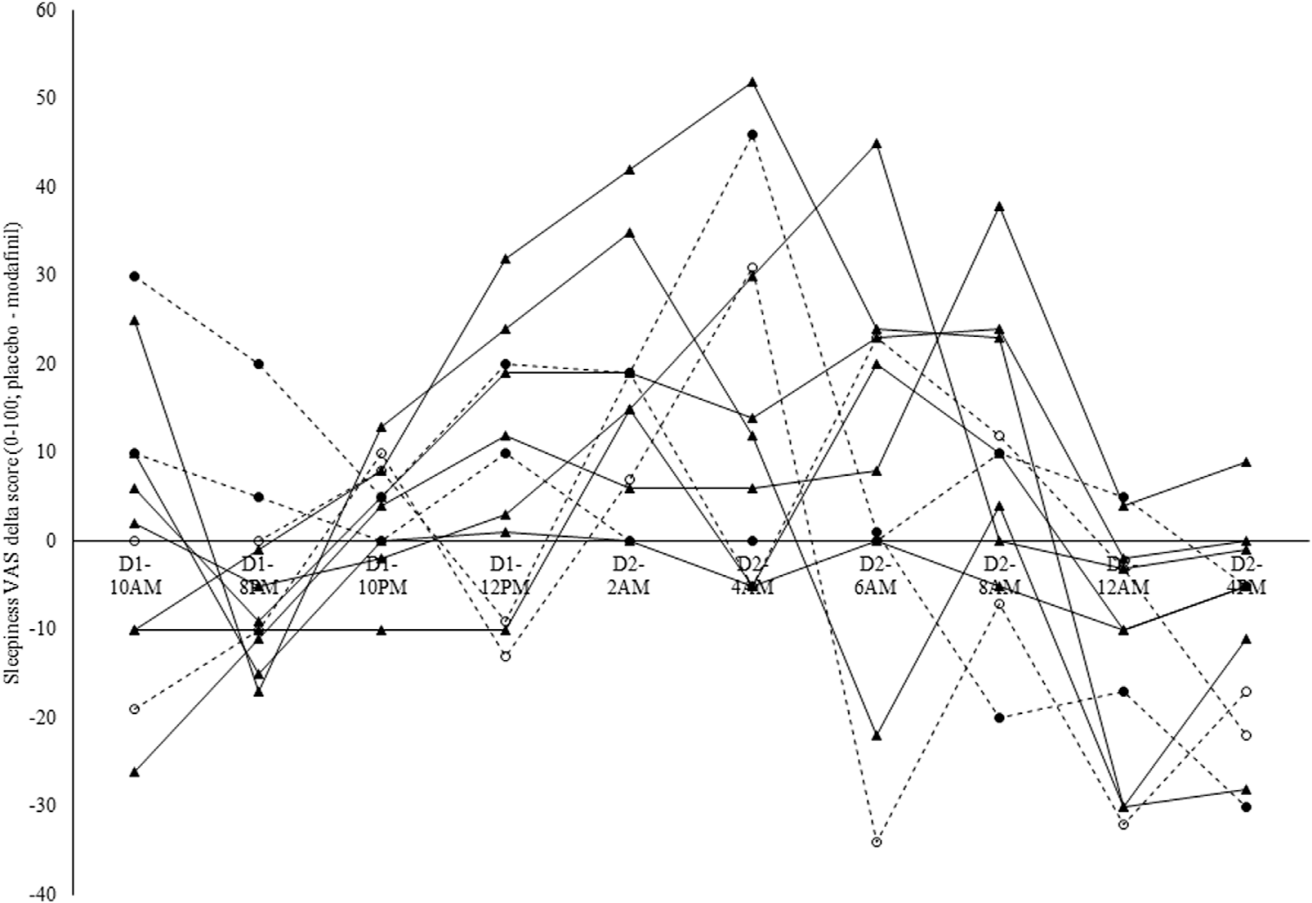
Individual modafinil effectiveness profiles in terms of subjective sleepiness. Sleepiness visual analog scale (VAS) delta scores were calculated by subtracting the sleepiness VAS score in the experimental condition (EXP-sleepiness) from the sleepiness VAS score in the control condition (CON-sleepiness). Each line represents one participant. Dashed lines indicate the participants that were genotyped for the catechol-O-methyltransferase (COMT) and Period Circadian Regulator 3 (PER3) single-nucleotide polymorphism (SNP). Full circles = C/C; Hollow circles = C/C. The intraclass correlation coefficient (ICC) for perceived sleepiness was 0.19.

Intraclass correlation coefficients were derived from the estimates for between- and within-subject variance in the delta scores (CON-EXP) in non-adjusted RT (between-subject variance = 1720; within-subject variance = 189) and sleepiness (between-subject variance = 18; within-subject variance = 77). The ICC for non-adjusted RT delta scores was 0.90 (see Figure 6), while for perceived sleepiness delta scores this was 0.19 (see Figure 7). Using the benchmarks suggested by Landis and Koch (1977), the sleepiness-ICC was slight (i.e., interindividual differences in the effect of modafinil on sleepiness are variable over time) and the non-adjusted RT-ICC was almost perfect (i.e., interindividual differences in the effect of modafinil on non-adjusted RT are robust over time).

### 3.7 Participant blinding method

All participants were able to correctly indicate when they took modafinil or slow-release caffeine (i.e., participants were still unaware that they did not take slow-release caffeine but a placebo instead).

### 3.8 Independent variables

#### 3.8.1 COMT and PER3 genotyping

Because only four participants were genotyped for the COMT and PER3 SNP, no statistical analyses were applied to these data and these results are presented for their descriptive and explorative value. In terms of the COMT gene genotype, two participants were genotyped as being homozygotes for the A allele (i.e., A/A; see the two lines with the full circle symbol in Figures 6, 7; see Table 5), while the other two participants were genotyped as being heterozygotes (i.e., G/A; see the two lines with the hollow circle in Figures 6, 7; see Table 5). In terms of the PER3 gene genotype, all four participants were genotyped as being homozygotes for the C allele (i.e., C/C).

**TABLE 5:** Impact of catechol-O-methyltransferase (COMT) gene genotype, Pittsburgh Sleep Quality Index (PSQI) score and Single-Item Chronotyping (SIC) profile on the effectiveness of modafinil in delta non-adjusted reaction time (RT) and delta subjective sleepiness (mean ± SD).

	COMT gene genotype	
	Group A (i.e., A allele homozygotes; n = 2)	Group B (i.e., heterozygotes; n = 2)
Delta non-adjusted RT (ms; CON-EXP)	79 ± 51	13 ± 13
Delta subjective sleepiness (au; CON-EXP)	5 ± 3	-3 ± -3
PSQI score	4 ± 1.4	4.5 ± 0.7
SIC profile	Profile 5	Profile 3 and 5
Chronotype score	ME = 17 ± 2.8 AM = 16 ± 1.4	ME = 17.5 ± 0.7 AM = 15.5 ± 2.1

	PSQI score	
	Group A (score < 5; n = 6)	Group B (score ≥ 5; n = 5)
Delta non-adjusted RT (ms; CON-EXP)	39 ± 49	16 ± 31
Delta subjective sleepiness (au; CON-EXP)	4 ± 6	3 ± 8
Chronotype score	ME = 19.5 ± 2.9 AM = 13.7 ± 3.3	ME = 19.2 ± 2.3 AM = 13.8 ± 3.4

	SIC profile	
	Group A (profile 5; n = 7)	Group B (all other profiles; n = 4)
Delta non-adjusted RT (ms; CON-EXP)	22 ± 48	40 ± 30
Delta subjective sleepiness (au; CON-EXP)	4 ± 6	3 ± 8
PSQI score	4.3 ± 2.3	3.5 ± 1.3
Chronotype score	ME = 18.7 ± 2.4 AM = 13.6 ± 3.9	ME = 20.5 ± 2.6 AM = 14 ± 1.6

#### 3.8.2 Subjective sleep quality

On average, participants had a global PSQI score of 4 ± 2, with only two participants having a score above 5 (i.e., the threshold that is used in clinical practice to define a ‘bad’ sleeper). The maximum global PSQI score was 7. To evaluate a role of subjective sleep quality in the efficacy of modafinil, independent samples t-tests were conducted on those two outcomes where the effect of modafinil was most notable [i.e., delta non-adjusted RT (CON-EXP) and delta subjective sleepiness (CON-EXP)]. However, the grouping variable did not significantly impact any of the mean delta scores nor did it impact the mean chronotype score (see Table 5).

#### 3.8.3 Chronotype

In terms of the chronotype score, the group mean for the ME-subscale was 19 ± 3 and for the AM-subscale 14 ± 3. On the SIC, seven participants chose for chronotype profile 5 (i.e., highly active type), one participant for profile 4 (i.e., daytime type), two for profile 3 (i.e., daytime sleepy type) and one for profile 2 (i.e., evening type). To evaluate a role for chronotype in the efficacy of modafinil, independent samples t-tests were conducted on those two outcomes where the effect of modafinil was most notable [i.e., delta non-adjusted RT (CON-EXP) and delta subjective sleepiness (CON-EXP)]. However, the grouping variable did not significantly impact any of the mean delta scores nor did it impact the mean PSQI or chronotype score (see Table 5).

## 4 Discussion

The most important findings of our study are: 1) The use of modafinil improved both PVT performance and subjective sleepiness throughout the extended wakefulness paradigm; 2) Modafinil effectiveness shows stable interindividual differences in PVT performance, but more variable interindividual differences for subjective sleepiness; 3) In terms of subjective sleepiness, a rebound effect of modafinil use was observed following the extended wakefulness paradigm, whereby higher subjective sleepiness was perceived with modafinil use than with placebo; 4) Regarding vital signs and perceived side effects, no side effects were observed in terms of heart rate, blood pressure, body temperature and respiration rate. However, five out of the eleven participants did indicate some side effects such as headache, diarrhea, light nausea, palpitations and nervousness at one or more time intervals; 5) Sleep diaries showed the recovery sleep was not impacted by modafinil.

### 4.1 PVT performance and subjective sleepiness

The present study provides additional evidence that, in a sleep deprived state, modafinil improves PVT performance and subjective sleepiness (Colzato and Mourits, 2017; Van Puyvelde et al., 2022). A positive effect of modafinil on PVT performance and subjective sleepiness was observed 6 h after taking in the first 200-mg dose (i.e., 2a.m. during the extended wakefulness paradigm). As such, the peak positive effects of modafinil use coincide with the peak negative effects of the extended wakefulness paradigm, rather than with the plasma concentration peak (i.e., ∼2.5 h after oral administration (Wong et al., 1999)) and corroborates findings from Wingelaar-Jagt et al. (2022b). The authors of the aforementioned study evaluated the effect of 200 mg modafinil on vigilance during a limited period of sleep deprivation compared to 300 mg caffeine and placebo, and found that the most notable effects occurred 4–6 h after administration, similarly to what was observed in our study (Wingelaar-Jagt et al., 2022a). The pharmacological effects of modafinil are known to be wake-promoting and, as such, set up the substance to be primarily valuable in a sleep deprived state. Modafinil is known to have multiple effects on different brain areas and neurotransmitter systems in the brain (Becker et al., 2022; Mereu et al., 2013; Minzenberg and Carter, 2008). Like amphetamine and methylphenidate, it inhibits dopamine reuptake, underlying its positive effects on human performance. Moreover, besides the dopaminergic effects of modafinil, several non-dopaminergic effects of modafinil have been described (e.g., increase of electrical neuronal coupling) that could further explain its efficacy as a wake-promoting and cognitive enhancing substance (Mereu et al., 2013).

### 4.2 Side effects of modafinil use

Building upon the research agenda put forward by Van Puyvelde et al. (2022), we emphasized the need to perform further rigorous research into the possible side effects of modafinil during off-label use. In terms of the well-known side effects (as described in the method section), the occurrence rate did not significantly differ between both conditions. In addition, modafinil-specific side effects were not assessed by participants to potentially impact safely flying an aircraft. In contrast, a clear trend was observed where participants found it safer to fly in the modafinil condition than in the placebo condition. Regarding the vital physiological signals (i.e., heart rate, blood pressure, body temperature and respiration rate) no impact of modafinil was observed either, confirming previously published research (Wingelaar-Jagt et al., 2022b). In terms of subjective sleepiness, a rebound effect of modafinil use was evidenced. At 12a.m. and 4p.m. following the extended wakefulness, a higher subjective sleepiness was perceived in the modafinil condition compared to the placebo condition. This rebound effect of modafinil on sleepiness did however not impact the self-reported recovery sleep. As such, this result confirms the previously reported results of Wingelaar-Jagt et al. (2022a), that self-reported recovery sleep is not impacted by modafinil use. Nonetheless, as is shown by research of Bodenmann and Landolt (2010) and Bodenmann et al. (2009b), it is important to further evaluate the impact of modafinil on recovery sleep by making use of polysomnography. Electroencephalographic recordings in humans already demonstrated that modafinil does not cause sleep rebound as compensation for its waking properties in the absence of sleep debt (Batéjat et al., 2006). However, in mice, Modafinil has been found to induce sleep rebound (Kopp et al., 2002). This warrants further investigation into a possible self-reported rebound effect of sleepiness after taking modafinil and the mechanism underlying this.

Furthermore, the possible presence of overconfidence was evaluated with a prospective (prediction) and retrospective (evaluation) of performance. Correlations with the overall PVT performance measure (i.e., non-adjusted RT) revealed that participants could predict and evaluate their performance with a higher accuracy in the placebo condition than in the modafinil condition. In the modafinil condition, participants overestimated their own performance. This result contrasts the findings of Li et al. (2020), who reported that confidence indices remained unaffected by modafinil use, but confirms the findings of Baranski et al. (2004) and Baranski and Pigeau (1997) and of Gurtman et al. (2008), that demonstrated that modafinil use impacts the self-monitoring capacity and results in overconfidence. However, in the modafinil condition of the present study, performance changes over time were much less pronounced than in the placebo condition. Therefore, due to a simple range effect, it was more difficult to predict and evaluate performance changes in the modafinil condition than in the placebo condition and self-monitoring capacity was challenged to a higher degree in the modafinil condition. Overall, the present results indicate that there may be overconfidence amongst the modafinil users, but it remains to be determined if this has the magnitude that it would be of clinical/operational relevance.

Some concerns surrounding the use of modafinil exist in applied settings, for example, in military and civilian aviation, as well as within multiple other professional contexts (e.g., shift workers). Specifically, concerns are raised for both the rebound effect in subjective sleepiness, and of the inaccuracy when predicting one’s own performance–especially considering the clear trend for participants to find it safer to fly in the modafinil condition than in the placebo condition. In the present study, no flying task was included to evaluate whether this perception would be backed up by objective flying performance data. However, the lower accuracy in predicting one’s own performance in the PVT task in the modafinil condition should be sufficient to warrant raising awareness on this matter. Further, it stresses the importance of conducting a baseline test to familiarize the person with the action of modafinil and its impact on their specific performance before taking modafinil during work-related tasks in a sleep-deprived context. Moreover, it questions whether training the ability to self-monitor performance when using modafinil should be advised. This concern is especially valid if modafinil is administered not only in a sleep-deprived context, but also when the circadian cognitive performance nadir occurs during the time window in which modafinil-associated overconfidence effects are expected (i.e., two to 4 hours post-dose; (Baranski and Pigeau, 1997)). Training the self-monitoring ability might be specifically important during this specific time window, when the self-monitoring ability is at the greatest risk of inaccuracy. In general, we emphasize caution with the application of modafinil, and the need for individual strategies to optimize its use.

### 4.3 Interindividual variability in modafinil-effectiveness

By experimental design, the current protocol involved variations in perceived sleepiness, mood and presence of daylight. Consequently, ICC values can be interpreted as evidence of both stability (across day and night) and robustness (across a varying psychological state). Eventually, interindividual differences in the effect of modafinil were stable and robust in terms of the effectiveness to counteract the increase in non-adjusted RT due to sleep deprivation.

This trait-like characteristic of modafinil effectiveness suggests a role for genetic influence and thus the opportunity to individualize modafinil use and dosing based on genotype. Within the present study, the non-adjusted RT of the individuals that were genotyped as being homozygote for the A allele of the COMT gene (i.e., A/A) was robustly positively influenced by modafinil use. In contrast, for the individuals that were genotyped as being heterozygote for the COMT gene (i.e., G/A), the non-adjusted RT was not or less impacted by modafinil use. In terms of sleepiness throughout an extended wakefulness protocol, no distinction could be made between the participants based on the COMT gene genotype. These results infirm our hypothesis. Individuals with the G/G COMT gene genotype have been shown to have higher amounts of cortical COMT protein and, in addition, their COMT proteins have a three-to fourfold higher enzymatic activity compared to individuals with the A/A genotype (Chen et al., 2004). As such, the G/G genotypes have been suggested to have lower dopaminergic signaling in the prefrontal cortex (Chen et al., 2004), and modafinil use has been found to be more effective in this specific genotype (Bodenmann et al., 2009a). This contrasting explorative evidence highlights, once again, that the complex genetic basis for functional effects can very rarely be reduced to one specific polymorphism in one specific gene. Moreover, it stresses the importance of disclosing an individual’s genetic predisposition in a deliberate manner and only when the scientific evidence for the genetics mediating the predisposition is robust. Otherwise, the expected physiological and psychological response profile on, for example, the use of modafinil, could be significantly impacted by placebo and nocebo effects (Turnwald et al., 2019).

The individualization of modafinil use and dosing is recommended in a military context in a situation of acute sleep loss. In an extended wakefulness paradigm of 24 h, stable interindividual differences in modafinil-effectiveness are present, an individualization-strategy would thus optimize modafinil use and dosing, and limit the occurrence of any unwanted side effects. Eventually, the individualization-strategy should be based on risk-benefit profiles in which the risks and benefits are determined in a baseline test. The risks that should be focused upon in these risk-benefit profiles are the side effects (e.g., headache, diarrhea) and future research should further investigate the trait-like aspects of the occurrence of these side effects when taking in modafinil. Based on the present study results, the benefit that should be focused on is the effectiveness of modafinil to counteract sleep deprivation-associated decrease in vigilance, as this effect of modafinil appears to be robust in time. The effectiveness of modafinil to counteract the sleep deprivation-associated increase in sleepiness was found to be much less robust in time and, therefore, is less suitable for creating risk-benefit profiles. The added value of baseline testing is further confirmed by recent research from Wingelaar-Jagt et al. (2023), in which it was shown that fatigue vulnerable individuals seem to benefit more from modafinil administration than fatigue resistant individuals. Therefore, despite the potential added value of genotyping (i.e., minimizing the time-cost of baseline testing), as long as there is insufficient evidence on the role of specific genes in the efficacy of modafinil or in the occurrence of specific side effects, we do not recommend using genetic profiling.

### 4.4 Limitations

Despite an *a priori* sample size calculation indicating that a total of 10 participants was needed, the inclusion of 11 military student pilots limited our statistical possibilities, for example, in terms of stratifying our cohort based on genotype, PSQI-score and/or chronotype. Nevertheless, these stratifications provided meaningful descriptive and exploratory information and added value. As mentioned in the results section, the participant-blinding method was unsuccessful. All participants were able to correctly indicate when they took modafinil or the placebo. However, this limitation was anticipated via introducing the study to the participants as a study on the effect of two (modafinil and slow-release caffeine) potentially performance-enhancing substances. Subsequently, they were debriefed and informed that the slow-release caffeine-condition was a placebo condition only after completing all trials. This precautionary measure resulted in the participants expecting positive effects in both the modafinil and placebo condition. The lack of a real slow-release caffeine condition is, of course, an extra limitation to the present study. In an operational scenario the choice will, probably, never be between administering modafinil or nothing–caffeine is almost always an option. However, tolerance to caffeine develops quickly and therefore modafinil has added value. To uncover the mechanisms underlying interindividual variability in modafinil effectiveness, research comparing the effects of modafinil to a placebo is essential. Additionally, in the present study, sleep was not assessed with the golden standard technique (i.e., polysomnography) and the physiological parameters were monitored via self-assessment. This limits the conclusions that can be drawn from these data (e.g., self-counting respiration rate creates an internal focus on breathing and will have an impact) and calls for further confirmation in future research.

### 4.5 Future research

Regarding the off-label use of modafinil in a military context, a rigorous evaluation of its impact on thermoregulation and exercise tolerance is highly necessary. For example, Roelands et al. (2008) demonstrated that the combination of exercise and acute bupropion (i.e., like modafinil, also a dopamine reuptake inhibitor) administration in the heat caused hyperthermia without any change in the perception of effort or thermal stress. The presence of a rebound effect in subjective sleepiness and overconfidence should also be focused upon in future research. The relevance of a modafinil-associated rebound effect in subjective sleepiness should be further evaluated via study designs that include 1) objective measurements of sleepiness (e.g., Multiple Sleep Latency Test) and 2) more frequent assessments to map exactly when rebound occurs and how long it lasts. The determination of a possible interaction between the circadian cognitive performance nadir and the modafinil-associated overconfidence effect, and the potential to counteract modafinil-associated overconfidence via training the self-monitoring ability when using modafinil, are both important future research aims. In addition, future studies should further evaluate the role of placebo effects in the efficacy of modafinil, especially when modafinil is used occasionally on a long-term basis, and expectancy effects are given more opportunity to occur. Moreover, future research on intra- and interindividual variability in modafinil effectiveness should consider the replicate crossover design that was put forward by Atkinson et al. (2018) to evaluate the variability in modafinil effectiveness in greater detail. Lastly, the complexity of the genetic basis for modafinil effectiveness should be taken into account by determining the mechanism of action of modafinil and, subsequently, genotyping as much functional polymorphisms that could impact this mechanism of action as possible (e.g., SNPs that impact the functioning of receptors, enzymes).

## 5 Conclusion

The present study provides additional evidence that modafinil improves PVT performance and subjective sleepiness during extended wakefulness. No significant side effects (i.e., perceived as job impairing) occurred due to modafinil. However, a negative rebound effect in subjective sleepiness and a lower accuracy in performance self-assessment was observed in those using modafinil. Stable interindividual differences were observed in the effectiveness of modafinil to counteract the decrease in vigilance due to sleep deprivation. This trait aspect of modafinil effectiveness merits the use of risk-benefit profiles, determined by baseline test results, to individualize and optimize the application of modafinil. Further research should focus on quantifying the extent to which modafinil-induced overconfidence and subjective rebound sleepiness actually constitute potential problems in operational environments (e.g., perhaps using war game simulations).

## Data Availability

Due to the military nature of the data (i.e., confidential information), the data presented in this study cannot be made publicly available. Data can be made available upon request with permission of the third party. To submit a request, please contact Jeroen.VanCutsem@mil.be.
